# Intensive longitudinal assessment of stress and stress-related concepts across a weight loss program: Protocol for a longitudinal ecological momentary assessment study

**DOI:** 10.1371/journal.pone.0357572

**Published:** 2026-09-10

**Authors:** Chelsea L. Kracht, Ann M. Davis, Udayan Apte, Madigan Snodgrass, Taylor Y. Collier, Jeffery J. Honas, John M. Jakicic

**Affiliations:** 1 Department of Internal Medicine, University of Kansas Medical Center, Kansas City, Kansas, United States of America; 2 Department of Pediatrics, University of Kansas Medical Center, Kansas City, Kansas, United States of America; 3 Center for Children’s Healthy Lifestyles & Nutrition, Children’s Mercy Hospital, Kansas City, Missouri, United States of America; Pennington Biomedical Research Center, UNITED STATES OF AMERICA

## Abstract

**Background:**

Fluctuations in daily stress and the inability to cope with this stress (i.e., coping) can negatively impact behavior and potentially weight loss. Longitudinal studies using ecological momentary assessment (EMA) of psychological factors and behavior may inform interventions that can be adapted in real-time to modify stress and stress-related factors to enhance weight loss. This study’s purpose is to examine variability in stress and stress-related concepts and weight loss during a behavioral weight loss program with the intent of this informing future interventions.

**Methods:**

Individuals with obesity (body mass index: ≥ 30 kg/m^2^) who are newly enrolled in an existing community-based behavioral weight loss programs will be recruited. Participants will complete four visits, to coincide with before program (week-0), early program (6-weeks), program mid-point (3-months), and program end (6-months). Each visit will include height, weight, and questionnaires related to chronic stress and coping, and weight-related behaviors. Participants will then complete at least 14-days of EMA, including one morning survey, four signal-contingent surveys during fixed 3-hour windows, and one night-time survey daily via a smartphone application. During these 14-days, participants will also complete device-based measures for physical activity and sleep and waking saliva collection to evaluate cortisol. The primary exposure is the difference (i.e., variability) in signal-contingent survey stress, coping, and affect at baseline. The primary outcome is weight change across 6-months. This study received initial startup in March 2024, and was registered in Clinical Trials in October 2024. The study began recruitment in March 2025 and anticipate completion in early 2027.

**Discussion:**

Findings may inform justification for the pursuit of an ecological momentary intervention focused on improving stress while engaging in a behavioral weight loss intervention to support behavior change and potentially weight loss.

**Trial registration:**

ClinicalTrials.gov NCT06668259

## Introduction

By 2030, over 50% of adults will have obesity based on current population-based trends [1]. Obesity prevalence can be reduced by individual-focused lifestyle interventions as these programs are generally effective at helping people lose weight [2,3]. However, some people who engage in these interventions lose less weight than others [4]. This difference in weight loss may be influenced by psychological factors, such as perceived stress, that play a key role in behavior change [4]. Intensive longitudinal assessment of these psychological factors and daily behavior can inform the design of adaptive interventions that can intervene in real-time to change stress and thereby behavior, improving weight loss success.

Fluctuations in daily stress and inability to cope with stress (i.e., coping) can negatively impact behavior, thus weight loss [5]. However, successfully assessing this relationship on the day-to-day level has been difficult. Ecological momentary assessment (EMA) methodology can address this limitation by assessing the antecedent (*what happened before the behavior*), the behavior, and the consequence (*what happened after the behavior*) pathway, thus evaluating the temporality of state and behavior. Accordingly, EMA methodology includes sending short surveys [6], via mobile application on phone (mHealth), on psychological measures and behavior context. Studies using EMA methodology found that momentary stress and related health constructs (e.g., affect) are correlated with cortisol levels [7] and other health parameters (e.g., blood pressure) [8]. These findings were demonstrated in an EMA study amongst 143 children (ages 8–12 years), where morning self-reported stress was negatively associated with same day moderate-to-vigorous physical activity (MVPA), but not salivary cortisol collected after wakening [9]. In addition to stress, a person’s affect (i.e., emotion and mood) may also impact their ability to be active thus lose weight. In a systematic review of EMA studies (*n* = 12), positive affect predicted subsequent physical activity, but not negative affect [10]. Therefore, one’s higher perceived stress, lower coping, and lower positive affect may hinder physical activity [10], lead to poor eating behaviors [11], and limit weight loss.

Investigating daily psychological factors and obesity-related behaviors using EMA across a weight loss intervention may inform creation of an adaptive intervention. Data collected from EMA methodologies can lead to adaptive mHealth interventions, including ecological momentary interventions (EMI) which provide in-the-moment support based on self-report or device feedback to prevent behavior relapse [12]. One possible option where EMA may be used to promote behavior change is physical activity. Engaging in physical activity can be used to cope with stress, suggesting an EMI could propose physical activity amongst low coping times, and promote weight loss [13]. Investigating these claims while an individual is undergoing behavior change (i.e., weight loss) may help identify if these behaviors are valuable focus points for future adaptive weight loss interventions.

### Objective and aims

The overarching aim of this study is to elucidate potential pathways of stress and stress-related concepts with subsequent health behaviors during a behavioral weight loss intervention. The primary exposure is the difference (i.e., variability) in signal-contingent survey stress, coping, and affect early in the program. The primary outcome is weight change across a behavioral weight loss program, and secondary outcomes are physical activity and sleep. We hypothesize that high variability in daily stress will be related to less weight loss across the initial (12-weeks) and full (26-weeks) program (Hypothesis 1a). Our second hypothesis is that high variability in daily stress will be related to less physical activity and sleep across the initial and full program (Hypothesis 1b). These hypotheses were constructed as higher variability and fluctuations aligns with past literature that with individuals who experience lower stressful daily events and who are able to cope consistently and easily with this daily stress may be more active and lose more weight compared to individuals experience daily changes in stress and are less able to cope or find it difficult to do so [5,14]. Finally, we hypothesize that high variability in coping and positive affect will be related to less weight lost across the initial and full program (Hypothesis 2).

## Methods

### Study design

The current study, intensive longitudinal assessment of stress and stress-related behaviors across a behavioral intervention or *INSTANT*, is a prospective observational cohort of individuals enrolled in a community-based weight loss program. The University of Kansas Medical Center Institutional Review Board (IRB) approved this study in May 2024 (IRB#:00160436) and registered with ClinicalTrials.gov in October 2024 (NCT06668259). The current protocol follows the reporting guidelines for EMA studies (See S1 Table). This study was approved by the local IRB prior to study implementation, and in compliance with US Federal Regulations and local IRB policies, this study undergoes annual IRB review with an extension provided on at least an annual basis.

### Participants, eligibility, and recruitment

This study seeks 50 individuals who are newly enrolled in a community-based weight loss program centered in a metropolitan area of a Midwest state. Participants will be recruited from an existing 6-month-long community-based weight management program hosted by the University of Kansas Medical Center, local Diabetes Prevention Programs and other behavioral weight loss programs in the local area that may range from 6 to 12-months long. These weight management programs typically include trained health educators, 1-hour weekly video meetings, group peer support, structured nutrition plan (low calorie diet), personalized activity recommendations, and behavior change modifications [15]. Prospective participants will be recruited at their initial consultation or orientation visit for their behavioral weight loss program. Flyers will also be presented to participants at their initial visit. Measures were planned to coincide with initial enrollment (week-0), early weight change (week-6), and later weight change measures (week-12, week-26).

To be included, participants must be newly enrolled or plan to enroll in a community-based weight management program, must be over 18 years of age, have body mass index (BMI) ≥30 m/kg^2^, own a smartphone and be willing to download the mobile application to receive text messages [16]. Participants who chose to cease their community-based weight management program involvement after enrolling in the INSTANT study can still participate in later assessments, improving upon past studies which only included individuals who completed their program [17,18]. Indeed, stress may impact continued engagement in a weight management program. Participants will be excluded for being currently prescribed FDA-approved medications for weight loss, treatment for a medical condition that could affect body weight, or impacting stress levels (e.g., antidepressants), a current psychological condition that is untreated, hospitalization for a psychological condition within the past 12-months, or not being on a stable dose of treatment for at least 6-months. Other exclusion criteria are not being fluent in writing or speaking the English language, mobility limitations so they are unable to perform physical activity, and a family member who was previously enrolled or is currently enrolled in the study.

### Procedures

#### Screening.

Interested participants will complete a web screening tool using a secure online platform [19]. If a participant qualifies but is not currently enrolled in a community-based weight loss program, staff will provide them with local options so they are able to enroll and meet inclusion criteria. Potential participants who met screening criteria will be contacted for an initial phone call, during which a trained staff member will inquire about the participant’s ability to travel to visits, wear an accelerometer on their wrist, complete a salivary cortisol kit and EMA surveys related to stress and sleep patterns as well provide their health history and current medication usage. Eligible individuals will be scheduled for their baseline visit (week-0).

#### Baseline (week-0).

In the first visit, written consent will be obtained from the participant prior to performing study procedures. Height and weight will be assessed by a trained staff member. The participant will complete questionnaires and will be fitted with an accelerometer to wear for 14-days. Participants will also be provided a 5-day salivary cortisol kits. The procedures for collecting, storing and transporting the saliva samples will be explained by trained staff in additional to the participant watching a short tutorial on the collection process and provided with written instruction. Participants will have the EMA app downloaded to their phone, and receive a tutorial on the app. Beginning the next day after the baseline visits, the participant will respond to EMA prompts for 14-consecutive days and wear the accelerometer. After 14-days, participants will return their accelerometer and saliva kit and delete the EMA app off their phone.

#### Follow-up visits (week-6, week-12, week-26).

At Visit 2–4, participants will have their height and weight measured, complete questionnaires, be provided the 5-day salivary cortisol collection kit, have an accelerometer fitted to their wrist to wear for 14 days and have the EMA app reinstalled on their phone to conduct the same EMA questions for 14-days. To reduce questionnaire burden in follow up visits, participants were given the option to complete questionnaires 1-week ahead of the visit using an online tool (REDCap). Harms and adverse events will be assessed at each visit. Study assessments are low-risk, thus we anticipate few study-related harms.

### Measures

**Fig 1:**
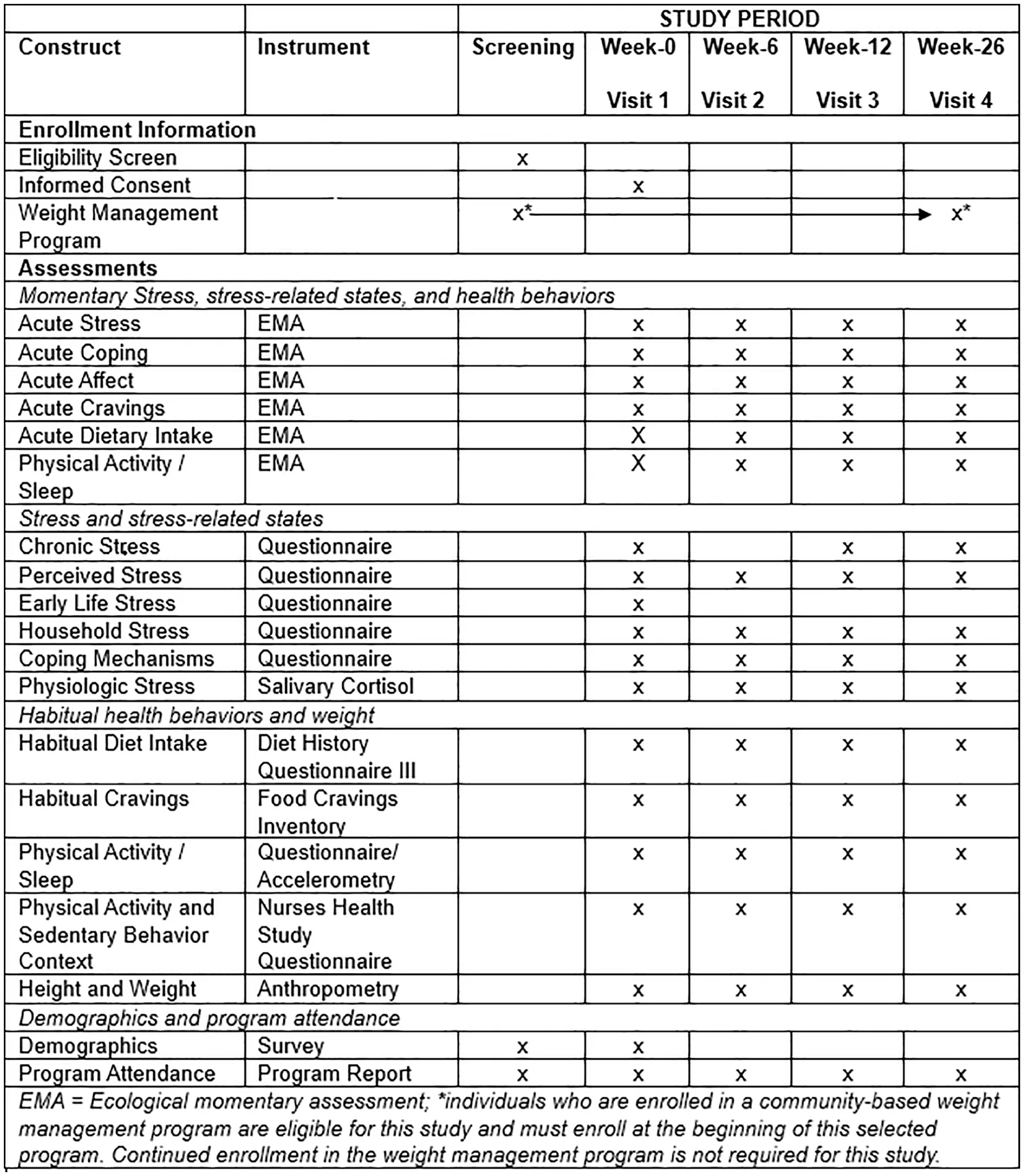
Schedule of enrollment, and assessments during study.

As shown in Fig 1, momentary stress, demographics, attendance, stress and related concepts, health behaviors, and weight will be collected across this study.

#### Ecological momentary assessment (EMA).

Participant’s current state and behaviors will be collected using a customizable software (LifePak, LifeData Corporation, Marion, IN). This software is compatible with both Apple and Android iOS delivered phone notifications as reminders, and study surveys will be completed within the app. Questions occur in the same order for all surveys and are provided as supplementary material (S2–S4 Tables) and a detailed account of constructs is provided in Fig 1. Participants will receive six surveys, including four signal-contingent and two fixed surveys (i.e., available for a fixed time period), through the app each day for 14 continuous days as performed by others measuring physical activity [20]. Signal-contingent surveys occurred randomly within fixed 3-hour windows (8:00am–11:00am, 11:00am-2:00 pm, 2:00 pm-5:00 pm, and 5:00–8:00 pm) and expire 45 minutes after the initial reminder. The two fixed surveys include the morning and evening survey; the morning survey will be available for four hours (8:00am-12:00 pm), and three hours for the evening survey (5:00–8:00 pm). These schedules may be shifted for non-traditional schedules, such as shift work, to ensure participants are available to answer these questions during their waking hours. Participants will be provided with three reminders for signal-contingent surveys, including the initial reminder, and reminders at 15-minutes and 30-minutes, if necessary. Participants will be sent reminders for the morning and evening surveys every hour, if necessary. Sampling time frames and prompt strategies were informed by past EMA studies [21,22]. Participants will be expected to complete at least one morning survey and two within-day surveys (~50%) for 5-days [23], with an additional incentive for 7-days in a row as conducted in our preliminary EMA studies.

#### Primary exposures.

**Variability in stress and coping:** In the morning survey, morning stress and coping will be assessed using questions based on the Daily Health Diary as assessed in past EMA studies [23,24], their own current stress and their perceived ability to cope with stress. Participants will be prompted to indicate the current stress, with response options ranging from 0–10 and 10 indicating higher stress. Participant coping will be queried with a similar question on their ability to manage stress right now, with the same response options and a higher score indicating a higher ability to manage stress.

**Variability in affect:** During signal-contingent surveys, participants will answer a set of five brief questions including positive and negative affective response. Then, participants will complete 5-questions based on how they are currently feeling positive and negative affect based on the Positive and Negative Affect Scale (PANAS), a validated scale which has been adapted in other EMA studies of affect [10,24,25]. These questions will be asked in the same order and are based on previous EMA studies [6,23,26], with response options ranging 1–4, with higher indicating more prevalent feelings of the emotion. An average of the two positive affect questions and three negative affect questions will be calculated. The average positive affect score will be compared for variability in affect.

#### Other stress-related states, and health behaviors.

As shown in Table 1, morning, signal-contingent, and evening surveys will assess other relevant considerations. The morning survey includes 16 questions, ending with the two questions that assess stress and coping. Participants will be asked, based on the previous night, how many hours of actual sleep they received, their bedtime and wake-time, and overall sleep quality. These questions are adapted from the Pittsburgh Sleep Quality Index, which is validated in adults [27]. This is followed by two questions on accelerometer and saliva compliance and then questions related to coping with stress and cravings the previous day. Cravings will be assessed using an adapted momentary scale of the Food Cravings Inventory (FCI). The FCI is questionnaire queries five different subscales including high fat foods, sweets, carbohydrates/starches, fast foods, and fruits and vegetables; this scale has been validated in adults [28]. FCI responses are via a 5-point scale, with higher scores indicating more frequent cravings. In the momentary question, we will ask how often they felt cravings for each of the 5-main items, with similar Likert scale responses. For example, they will be asked how frequent they craved carbohydrates/starches during the day. They will be asked if they indulged in cravings, and if so, which of the five craving groups with check box option.

**Table 1:** Daily Schedule of Ecological Momentary Assessment Surveys sent to participants for 14-days.

Construct	Morning Survey (8a-12p)	Period 1 (8a -11a)	Period 2 (11a -2p)	Period 3 (2p-5p)	Period 4 (5p-8p)	Evening Survey (6p-10p)
Sampling	Fixed	Random	Random	Random	Random	Fixed
*Stress and stress-related concepts*						
Acute Stress	x	x	x	x	x	x
Coping Mechanisms	x					x
Affect		x	x	x	x	
Chaos/ Disorder		x	x	x	x	x
Context		x	x	x	x	
*Health Behaviors*						
Diet	x					x
Cravings	x					x
Physical Activity						x
Sleep	x					
Survey Burden						x

Participants will also be surveyed about accelerometer wear at night and salivary cortisol collection in the morning survey, and accelerometer wear during the day will be queried in the evening survey.

The signal contingent survey includes eight questions total. First, participants will be asked a 1-question item to assess routine and disorder within the moment adapted from the Confusion, Hubbub and Order Scale [29]. Then, participants will complete the PANAS scale as described previously. Finally, participants will be queried where they are located (e.g., home, work) and who they are with (alone, friends, etc.) to address context.

The evening survey includes 11 questions. First, participants will report overall daily stress, and then indicate transient stressors, including home, school, job, conflicts with partner or children, and financial stress using checklist options [30]. This will be followed with four questions related to home chaos that demonstrated high validity and reliability in a previous adult sample (Cronbach’s alpha = 0.79, re-test reliability, r = 0.82) [31]. Then, participants will be asked if they engaged in moderate to vigorous physical activity, and if so, how many minutes during the day. These questions were based off primary questions the Godin-Shephard leisure questionnaire which has been validated in adults [32]. Survey burden will be assessed using a 1-item question at the end of the survey. Participants will be queried to the burden of completing the survey that day, with a scale of 1–5, with a higher score indicating it was very burdensome.

#### Stress-related states and health behaviors.

Momentary measures, demographics and attendance, stress and related states, and health behaviors will be assessed across Visits 1–4 (Table 1).

#### Primary outcome: Weight.

Participants’ height (cm) and weight (kg) will be objectively measured by a trained research assistant using standard operating procedures, such as shoes removed, with a mounted stadiometer and portable scale. Measurements will be taken in duplicate and an average was used. If the measurements differed by more than 0.5 units, then a third measurement was taken. Body mass index (BMI, weight/height^2^, kg/m^2^) will be calculated. Weight at Visits 2−4 will be subtracted from Visit 1 (Week-0) to calculate change in weight.

#### Secondary outcome: Physical activity and sleep.

Physical activity will be using an Actigraph GT3X+ accelerometer (Actigraph, LLC, Pensacola, FL) recorded at 50hz sampling rate and placed on the wrist of the nondominant arm for the entirety of the EMA protocol, including overnight. The GGIR algorithm will be used to detect non-wear time then equate remaining time as sleep by wear angle [33]. Days with >10 hours of awake wear and ≥20 hours of total wear time (awake and sleep) will be considered a valid day. GGIR version 3.2.6 will be used to process metrics using the remaining raw accelerometer data [33–35]. The Euclidian Norm Minus One (ENMO), the vector magnitude of three axes minus 1 g will be calculated and used to classify physical activity level. Activity will be classified as inactive (0–39 mg), light ([LPA], 40 mg ≤ ENMO <100 mg), and MVPA (ENMO ≥100 mg) [36]. Due to possible random wrist movement, bouts of ≥1 minute where 80% of the activity was classified as MVPA were used as performed by others [37]. Variables will be summarized for each participant for available days (minutes/day). In addition to device-based measures, a best practice is to also include questionnaires [38]. For physical activity intensity, participants will also complete the Godin-Shephard Leisure Time Physical Activity Questionnaire to assess physical activity within the past week. This questionnaire is validated within adults [32]. As for sleep, participants will report their recent sleep habits using the Pittsburgh Sleep Quality Index, which is validated in adults [27]. Participants will report sleep quality, sleep duration, and daytime dysfunction.

#### Exploratory outcomes.

Stress will be assessed at the individual level (chronic, current, and early life exposure) and at the home level (current household chaos). Chronic stress will be assessed as both stressful life events, and overall stress in the past month. First, the Brief Life Event Questionnaire will be used to assess chronic stress each 3-months (Visit 1, Visit 3, Visit 4). This questionnaire is a self-report measure to quantify presence of potentially traumatic and stressful events (e.g., close family member died, major financial crisis), and whether they still think about the event a lot, a little, or not at all [23,30]. Specifically, participants will be surveyed about the presence of each event within the last 6-months, or since they completed the questionnaire. This questionnaire has demonstrated high test-retest reliability (kappa: 0.88–1.0), and high sensitivity and specificity for the previous 6-months (0.89, 0.74, respectively) [39]. Second, recent stress in the past month will be assessed using the 10-item Perceived Stress Scale. This is a scale a questionnaire about feelings and thoughts over the last month, and has demonstrated in adults high values in metrics of validity and reliability [40]. Third, the early life exposure to stressful events, especially during critical periods of growth, may impact child development and later health [41,42]. We will assess adverse child experiences (ACEs) through 11 questions which query participants about events that happened prior to age 18 years old. These queries will be about family dysfunction (e.g., family member with mental illness, member in prison), and abuse (physical, verbal, etc.), and are adapted from the Behavioral Risk Factor Surveillance System ACE module, which is conducted nationwide in adults [43]. This scale’s validity and reliability was recently examined using measurement invariance, and demonstrated the total scoring is appropriate for analysis [44]. Total number of ACEs will be a potential mediator in analysis. Finally, the Confusion, Hubbub, and Order Scale (CHAOS) is a 15-item measure assessing disturbance, organization, and noise levels in the home. It has been validated in parents of young children to assess the home environment [29], and will be assessed at each visit.

Participants will be queried about using coping mechanisms, specifically “*What have you done to cope with your stress in the past 30-days?*” Participants have 12-options, including mindfulness practices, social support, sedentary behavior, and other options. These questions are based on the National Institutes of Health Effects of COVID-19 Outbreak – Adult Questionnaire for stressful events related to COVID-19; PhenX developed response options from crowdsourced to enable rapid response and address majority of coping practices [45,46]. Use of coping mechanisms, and specific coping mechanisms used will be exploratory variables.

Physiologic stress will be assessed through salivary cortisol. Participant will be provided with collection tubes at each visit, to collect 5-days of cortisol. Participants are asked to collect saliva on the first five days to coincide with physical activity and EMA collection. Five days was selected to correspond with other protocols and reduce participant burden [9]. Participants will be asked to collect a sample of their saliva within 1-hour of waking up, around the same time at all time points, and prior to consumption of sustenance. Participants will log their wake-up time and exact time of sample collection on a sheet and place the collection tube in a freezer until the end of the EMA protocol (~14-days), and the participant will transport the frozen items to the lab in a cold container (Freezebox, Crystal Industries, Addison, TX). These samples will then be frozen until analysis. The cortisol will be analyzed using an ELISA-based immunoassay, which has a sensitivity of 0.111ng/mL and an assay range of 0.2–10 mg/ml for saliva, and a reliable intra and inter assay prediction (0.41, 0.58, respectively) [47]. Samples collected outside of the 1-hour waking window will be removed from analysis.

Habitual dietary intake will be assessed using a food frequency questionnaire, the Diet History Questionnaire III (DHQ), at each of the visits. The DHQ is a web-based system developed by the National Cancer Institute to administer food frequency questionnaires, and has been validated in adults [48]. This food frequency questionnaire will 135 common foods and beverages they consumed over the past month, and include portion sizes. The DHQ III information will be used to calculate Healthy Eating Index 2015 (HEI). HEI is a calorie-adjusted measure based upon meeting the Dietary Guidelines for Americans, and ranges from 0–100 with a higher score indicating better healthier dietary quality [49].

Habitual food cravings will be assessed by the FCI full form, which assess the cravings for specific types of foods over the past month. The FCI is described previously in the Ecological Momentary Section, but in brief the FCI is a 28-item questionnaire queries five different subscales including high fat foods, sweets, carbohydrates/starches, fast foods, and fruits and vegetables. FCI responses are via a 5-point scale, with higher scores indicating more frequent cravings [28]. High FCI scores have been negatively associated with sleep metrics in other samples [50].

Physical Activity and sedentary behavior context will be assessed using the Nurses Health Survey for Physical Activity questionnaire, a reliable and validated questionnaire [51]. Participants will be asked based on the past year or since they last completed the questionnaire, on average how much time they spent in various activities per week and number of days they exercised. There were ten categorical responses, increasing from minutes (0, 1–4, 5–19, 20–59) to hours (1, 1–1.5, 2–3, 4–6, 7–10, and 11+). In a separate section of the questionnaire, participants were asked how many hours per week they spent sitting (options: away-from-home, watching television/ streaming [herein: screens], other), and standing/walking (options: at-work, at-home), with similar categorical responses. Responses will be multiplied by appropriate conversion factors to calculate minutes/week.

#### Demographics and program attendance.

Participants will report demographic characteristics (e.g., age, race/ethnicity, income) and previous weight loss history at baseline. Enrollment in the program will be queried from the respective programs at initial screening to confirm meeting inclusion criteria. Final session attendance dates will be queried from the program at end of study.

#### Stipends and incentives.

Participants will be paid up to 300 USD for completing the study, including 50 USD for each visit. Participants will also be eligible for a reduce initiation fee for the existing 6-month-long community-based weight management program hosted by the University of Kansas Medical Center. For completing Visit 1, participants will be given a wearable device (estimated value 100 USD). These devices can be used during the accelerometer wear periods. However, participants will be told to prioritize wearing the accelerometer during the selected periods.

### Analytical approach

#### Aim 1: Examine the association between variability in individual stress and weight change.

For hypothesis 1a, that high variability in daily stress will be related to less weight loss, linear regression will examine week-0 average and change frequency in daily stress (morning survey) with weight change at week-12 (visit 3) and week-26 (visit 4). For hypothesis 1b, that high variability in daily stress will be related to less physical activity and sleep, multi-level models with logit link function (and binomial distribution) will be used to examine association between week-0 morning stress and same day MVPA and sleep, with an interaction of time/day*morning stress. For exploratory analyses, we will examine the associations of daily stress with MVPA and sleep at other time points (week-6, week-12, and week-26), lagged day effects of stress (i.e., stress on day 1 influencing MVPA and sleep on day 2) to preserve temporal ordering and considering inverse probability weighted marginal structural models, and the outcome of weight change at week-6.

#### Aim 2: Examine the association between variability in coping and positive affect and weight change.

To address hypothesis 2, that high variability in coping and positive affect will be related to less weight loss, linear regression will examine week-0 average and change frequency in coping (morning survey) and positive affect (within day survey) with weight change at week-12 and week-26.

Potential covariates for both models include number of ACEs, program compliance (i.e., number of sessions attended), coping mechanisms (number and type), age, and sex. Statistical significance will be set at *p* = 0.05. All available data will be analyzed with a sensitivity analysis for individuals with complete data. Significant weight loss will be seen at 12-weeks (12.7 ± 19.9 kg), and 6-months (12.7 ± 35.2 kg) based on past studies in our community-based weight loss program allowing us to compare weight loss in Hypothesis 1a and 2. [15]

#### Sample size and power calculation.

Based on past EMA work (*n* = 39), there was an association between daily morning stress and feeding practices (*p* = 0.01) [52]. Considering physical activity and sleep are also impacted by stress [10,11], we anticipate being able to detect an association between stress variability with physical activity and sleep with prescribed amount (*n* = 50). This amount will allow us to compute a random linear regression model with 0.80 power, 0.3 effect size, 0.01 alpha error probability, 0.27 lower critical R^2^, and up to five predictors (such as potential covariates) [53]. Meeting minimum compliance (5-days), we would still have ≥5 observations/participant (≥250 observations) to calculate variability and detect meaningful associations.

#### Recruitment plans.

We will utilize existing connections with various community-based weight management programs across the selected area to ensure recruitment amount is achieved. Retention will be promoted with bi-weekly contacts between Visits 1–3, and monthly contacts between Visit 3 and 4. We will promote shorter visits for Visti 2–4 by providing surveys ahead of time for participants to complete via secure weblink, thus shortening time for weight, EMA and saliva set up at the visit. All available data will be sought in the case of discontinuation after Visit 1, with the priority of weight, EMA, surveys, and then saliva collection.

#### Data management plan.

All participants will be given an ID as part of the screening process. This ID will be stored on a secure database at the University of Kansas Medical Center. Only approved project staff will have access of the server. At each major time point (week-0, 6-weeks, 12-weeks, and week-26), we will collect other participant data including anthropometry (weight and height), accelerometry, questionnaires related to participant behaviors, stress, and stress-related states. Program attendance data will be collected after Visit 4. These values will be reported as tabular data and stored in a secure electronic data capture system. All subject-level clinical data will be preserved and shared. Shared data will be deidentified, and the original data will be maintained at the investigator’s institution. Completed salivary tubes will be frozen until analysis and stored at a biospecimen freezer at the local site. This data will be processed within 1-year of collection to calculate salivary cortisol, and remaining biospecimen will not be stored.

Missing data and data quality will be assessed throughout the study, including REDCap validation rules, double data entry of values (e.g., height and weight), reviewing participant salivary collection logs, and periodic review of study records. For EMA, participants are contacted multiple times during their use period to ensure compliance and any technical issues that may impact obtaining data. For accelerometry, device non-wear periods and implausible values will be identified through reviewing device wear logs and routine quality control procedures of devices. During analysis, we will examine patterns of missing data. If appropriate, mixed models will be used; these models are robust to incomplete repeated measures under a missing-at-random assumption. Further, sensitivity analyses may be considered to evaluate the impact of missing data on study findings.

#### Current progress.

The current study began recruitment on March 7^th^, 2025. As of January 2026, we have enrolled nine participants, six participants have completed their week-6 visit, and four have completed their 3-month visit. One person was lost to follow up after their 3-month visit. No adverse events have been reported. We anticipate concluding recruitment in 2026, with data collection completed by February 28^th^, 2027. Descriptive and final results are expected to be published in the year 2027.

## Discussion

This study aims to examine potential pathways of stress and stress-related concepts with subsequent weight change and health behaviors during a behavioral weight loss intervention. Utilizing mHealth technology, assessing various forms of stress and through multiple modalities, and assessments in parallel to a behavioral weight loss program may help improve our understanding from cross-sectional or singular stress measures. The resulting data will inform if a much-needed EMI can support individuals as they engage in behavioral weight loss program in the real-world for lasting impact. Pending study findings, there are three main areas for future exploration. First, future studies may include examining similar constructs and outcomes amongst individuals using obesity-management medications; these medications are becoming more common [54] but individuals using these medications are excluded from enrolling in this study. Second, other considerations for future study may be examining other mental health constructs (e.g., depressive symptomology). The current study utilizes multiple questionnaires that are highly correlated with depressive symptoms (e.g., Perceived Stress Scale [40]), but does not directly address this construct. Finally, consideration of amount, types, and use of coping mechanisms may still require further exploration. It is possible that an individual with high coping ability, and thus lower variability, may still result in positive health behavior (e.g., physical activity and adequate sleep) [5,8]. Use of coping mechanisms may also improve as individuals engage in a behavioral weight loss program [5]. The current study addresses this concern by collecting additional details on coping mechanisms used at the individual and daily level.

We will present our findings at local meetings and community events, including at the community-based weight management program at University of Kansas Medical Center. These efforts will increase awareness of the importance of stress and coping while making a behavior change, but also opportunities to change the home and local to facilitate adequate physical activity and sleep. We will present this information at the appropriate health literacy level and create informational flyers for adults and communities to utilize this information amongst their community. We will prepare a lay person handout of results and next steps to share with community partners who helped with recruitment, along with the flyers. The results from our study will have implications to identify potential pathways to improve stress and mental health for long-term behavior change and weight management success.

## Supporting information

S1 Table Checklist for Reporting Ecological Momentary Assessment Studies. (DOCX)

S2 Table Morning Survey Questions, Answers, and Descriptions. (DOCX)

S3 Table Evening Survey Questions, Answers, and Descriptions. (DOCX)

S4 Table Within day Survey Questions, Answers, and Descriptions. (RTF)

S1 Protocol INSTANT Protocol. (PDF)

S1 Checklist SPIRIT 2025 Checklist. (DOCX)

S2 Checklist PLOE Human Subjects Research Checklist. (DOCX)

## Abbreviations

BMI: Body Mass Index
ELISA: Enzyme-linked immunosorbent assay
ENMO: Euclidian Norm Minus One
FCI: Food Cravings Inventory
FDA: Federal Drug Administration
EMA: Ecological Momentary Assessment
EMI: Ecological Momentary Intervention
MVPA: moderate-to-vigorous physical activity
PANAS: Positive and Negative Affect Scale
