# Supplementary material for: Intensive longitudinal assessment of stress and stress-related concepts across a weight loss program: Protocol for a longitudinal ecological momentary assessment study
Source: PLoS One. 2026 Sep 10;21(9):e0357572. doi: 10.1371/journal.pone.0357572 (PMC13561333; doi:10.1371/journal.pone.0357572)
Supplement: Supplementary file 1 [file pone.0357572.s005.pdf]

Intensive longitudinal assessment of stress and stress-related concepts across a behavioral weight loss intervention: INSTANT Study

PRINCIPAL INVESTIGATOR:
Chelsea Kracht
Department of Internal Medicine, Division of Physical Activity and Weight Management
913-588-1655
Email: ckracht@kumc.edu

Co-Investigators:
Ann Davis, PhD, Department of Pediatrics
John M. Jakicic, PhD, Department of Internal Medicine

VERSION NUMBER/DATE: Version Number 7/November 24, 2025

REVISION HISTORY

Revision #	Version Date	Summary of Changes	Consent Change?
1	10.2.24	Minor updates	Yes
2	11.7.24	Minor updates	Yes
3	12.13.24	Minor updates	Yes
4	3.6.25	Minor updates	Yes
5	6.20.25	Minor updates	Yes
6	11.3.25	Minor updates	Yes
7	11.24.25	Minor updates	Yes

Table of Contents

1.0 Study Summary 2
2.0 Objectives* 2
3.0 Background* 2
4.0 Study Endpoints* 2
5.0 Study Intervention/Investigational Agent 4
6.0 Procedures Involved* 4
7.0 Data and Specimen Banking* 9
8.0 Sharing of Results with Subjects* 10
9.0 Study Timelines* 10
10.0 Inclusion and Exclusion Criteria* 10
11.0 Vulnerable Populations* 11
12.0 Local Number of Subjects 11
13.0 Recruitment Methods 11
14.0 Withdrawal of Subjects* 12
15.0 Risks to Subjects* 12
16.0 Potential Benefits to Subjects* 12
17.0 Data Management* and Confidentiality 12
18.0 Provisions to Monitor the Data to Ensure the Safety of Subjects* 14
19.0 Provisions to Protect the Privacy Interests of Subjects 14
20.0 Compensation for Research-Related Injury 14

21.0 Economic Burden to Subjects 14
22.0 Consent Process 14
23.0 Process to Document Consent in Writing 13
24.0 Setting 15
25.0 Resources Available 15

1.0 Study Summary

Protocol Information	Description
**Study Title**	INSTANT Study
**Study Design**	Prospective Observational Cohort
**Primary Objective**	Examine the association between variability in individual stress and related concepts with weight loss, physical activity, and sleep across a behavioral weight loss program
**Study Population**	Individuals who are enrolled in a community-based weight loss program centered in a metropolitan area of a Midwest state will be eligible to participate
**Sample Size**	50
**Study Duration for individual participants**	6-months (26-weeks)
**Study Specific Abbreviations/ Definitions**	EMA: Ecological Momentary Assessment

2.0 Objectives

Specific Aim 1: examine the association between variability in individual stress and weight loss across a behavioral weight loss program.

Hypothesis 1a: High variability in daily stress will be related to less weight loss across the initial (3- months) and full (6-months) program.

Hypothesis 1b: High variability in daily stress will be related to less physical activity and sleep across the initial and full program.

Specific Aim 2: Examine the association between variability in coping and positive affect, and weight loss across the behavioral weight loss program.

Hypothesis 2: High variability in coping and positive affect will be related to less weight lost across the initial and full program.

3.0 Background

Background: By 2030, recent estimates find that over 50% of adults will have obesity.[1] Obesity prevalence can be reduced by individual-focused lifestyle interventions as these interventions are generally effective at helping people lose weight.[2,3] However, some

people who engage in these interventions lose less weight than others.[4] This variability in weight loss may be influenced by psychological factors, such as perceived stress, that play a key role in behavior change.[4] Intensive longitudinal assessment of these psychological factors and daily behavior can inform the design of adaptive interventions that can intervene in real-time to change behavior and improve weight loss success.

Fluctuations in daily stress and ability to cope with this stress (i.e., coping) can negatively impact behavior, thus initial weight loss.[5] However, successfully assessing this relationship on the day to day level has been difficult. Ecological momentary assessment (EMA) methodology can address this limitation by assessing the Antecedent (*what happened before the behavior*)→ Behavior → Consequence (*what happened after the behavior*) pathway, assessing the temporality of state and behavior. Accordingly, EMA methodology includes sending short surveys,[6] including via mobile application on phone (mHealth), on psychological measures and behavior context. Studies that employed EMA methodology found momentary stress and related health constructs (e.g., affect) are correlated with cortisol levels[7] and other health parameters (e.g., blood pressure).[8] These findings were demonstrated in an EMA study amongst 143 children (ages 8-12 years), where morning self-reported stress was negatively associated with child moderate-to- vigorous physical activity (MVPA) that same day, but not salivary cortisol collected after wakening.[9] Other than stress, a person's affect (i.e., emotion and mood) may also impact their ability to be active thus lose weight. In a systematic review of EMA studies (*n*=12), positive affect predicted subsequent physical activity, but not negative affect.[10] Therefore, one's higher perceived stress, lower coping, and lower positive affect may hinder physical activity[10] and poor eating behaviors[11] and subsequently lose weight.

Investigating daily psychological factors and behavior using EMA across a weight loss intervention is the first step to creating an adaptive intervention. Data collected from EMA methodologies can lead to adaptive mHealth interventions, including ecological momentary interventions (EMI) which provide in-the-moment support based on self-report or device feedback to prevent behavior relapse.[12] One example that may be used in an EMI for behavior change is physical activity. Engaging in physical activity can be used to cope with stress, suggesting an EMI could propose physical activity amongst low coping times, and promote weight loss.[13] Investigating these claims while an individual is undergoing behavior change (i.e., weight loss) may help identify if these behaviors are valuable focus points for future adaptive weight interventions.

Preliminary Studies:

A thorough assessment of stress-related constructs[4,14] and obesity-related behaviors[15] across multiple time points may develop a tailored adaptive intervention. Accordingly, there is limited assessment of stress-related concepts and obesity-related behaviors during weight loss or life transitions in general (e.g., retirement). The sole EMA study during a weight loss intervention was conducted only women with overweight and obesity (*n*=60) amongst two assessments periods (baseline and 12-weeks, end of study).[16] In this study, they found women who felt less anxiety after overeating were more likely to lose less weight at week-12.[16] At this same timepoint, women (*n*=50) ate less after higher than usual stress, if they had engage in more physical activity.[17] These results propose that stress is important to subsequent health behaviors after weight loss, but these relationships in the intermediary period (e.g., early weight loss) is unknown. Proposed next steps from this study were to investigate stress variability, coping, and how these both impacted health behaviors.[16,17] These two gaps will be fulfilled by the proposed study. Another two gaps the proposed study will fill are including males (not represented in the past study) and early weight loss, which is important to weight loss success.[4]

Specific to the EMA protocol, we conducted a pilot study with parents of young children (*n*=30) for similar completion (1-morning, and 2 within-day) and survey burden. All parents contributed at least 2-days. Importantly, this high completion allows us to examine variability within-day and across days, which are our primary predictors in the proposed study. To achieve this compliance, best practices for conducting EMA methodology were used.[18,19] We prompted participants ≤6 times/day, included an incentive for completing the requested number of surveys (i.e., "complete days"), and consecutive days (7-days in a row): 76% participants completed at least 7+ consecutive days in a row, and 93% achieved 5 days. In addition to appropriate prompting, a research assistant contacted participants within 2-3 days of the EMA procedure if there is low compliance and conducted trouble shooting with the participant. Participants were also contacted 5-days, and 7-9 days into the EMA procedure to report acquired incentives gained and encourage continued participation.

The overarching aim of this study is to elucidate mechanisms of stress and stress-related concepts with subsequent health behaviors during a behavioral weight loss intervention. These relationships will be investigated using EMA methodologies on daily behaviors, which can then inform an EMI of an adaptive intervention to support existing individual weight loss programs in a R01 application. To thoroughly assess the mechanism, this study will conduct EMA measures at multiple timepoints within a set of diverse participants engaged in a community-based weight management program.

4.0 Study Endpoints

- Primary outcome:
    - Hypothesis 1a: weight loss at initial (12-weeks), and full program (26-weeks)
    - Hypothesis 1b: subsequent MVPA and sleep (same day)
    - Hypothesis 2: weight loss at initial (12-weeks), and full program (26-weeks)
- Secondary outcomes include early weight loss (6-weeks),
- Exploratory analysis: we will examine change score within the day (within day survey differences), the lagged day effects of stress (i.e., stress on day 1 influencing physical activity and sleep on day 2).

5.0 Study Intervention

This study is a prospective observational study, so it does not include a study intervention. However, participants will be enrolled in a community-based behavioral weight loss program. This program includes trained health educators, 1-hour weekly video meetings, group peer support, structured nutrition plan (very low calorie diet), personalized activity recommendations, and behavior change modifications.

6.0 Procedures Involved

We will conduct prospective observational study with measures at baseline (week-0, Visit 1), early weight loss (week-6, Visit 2), mid program (12-weeks, Visit 3), and end of program (26-weeks, Visit 4).

As shown in Table 1., where momentary stress, demographics, attendance, stress and related concepts, and health behaviors, and weight will be collected across this study.

**Table 1. Constructs Assessed during Study**					
**Construct**	**Instrument**	**Week-0**<br><br>**Visit 1**	**Week-6**<br><br>**Visit 2**	**Week-12**<br><br>**Visit 3**	**Week-26**<br><br>**Visit 4**
Momentary Stress, stress-related states, and health behaviors					
Acute Stress	EMA	X	x	x	x
Acute Coping	EMA	X	x	x	x
Acute Cravings	EMA	X	x	x	x
Acute Dietary Intake	EMA	X	x	x	x
Physical Activity / Sleep	EMA	X	x	x	x
Demographics and Attendance					
Demographics	Survey	x			
Program Attendance	Program Report		x	x	x
Stress and related states					
Chronic Stress	Questionnaires	x		x	x
Early Life Stress	Questionnaire	x			
Household Stress	Questionnaire	x	x	x	x
Coping Mechanisms	Questionnaire	x	x	x	x
Physiologic Stress	Salivary Cortisol	x	x	x	x
Habitual Health Behaviors and weight					
Habitual Diet Intake	DHQ	x	x	x	x
Habitual Cravings	FCI	x	x	x	x
Physical Activity / Sleep	Questionnaire/ Accelerometry	x	x	x	x
Physical Activity and Sedentary Behavior Content	NHS Questionnaire	x	x	x	x
Weight	Anthropometry	x	x	x	x

EMA= Ecological momentary assessment

- Momentary stress, stress-related states, and health behaviors:
  - *Acute Stress and Coping (morning survey):* Morning stress and coping will be assessed using questions based on the Daily Health Diary,[20] their own current stress and their perceived ability to cope with stress. Participants will be prompted "*On a scale from 0-10, with 0 being not stressed at all and 10 being very stressed, how would you rate your level of stress RIGHT NOW*", with response options ranging from 0-10 and 10 indicating higher stress. Participant coping will be queried with a similar question ("*On a scale from 0-10, with 0 being ineffective and 10 being effective, how would you rate your ability to manage stress RIGHT NOW?").*
  - *Acute Chaos, Stress, Affect, Context (within day survey):* Participants will be asked a 1-question item to assess routine and disorder within the moment adapted from the Confusion, Hubbub and Order Scale.[21] Then, participants will complete 5-questions in the same order related to how they are currently feeling positive and negative affect based on the Positive and Negative Affect Scale, as used in other EMA studies of affect.[10,20] Finally, participants will be queried where they are located (e.g., home, work) and who they are with (alone, friends, etc.) to address context.
  - *Acute Stress and Coping (end of day*): During the end of day survey, participants will report overall stress (i.e., "*Overall, how stressful was your day*?") with response options ranging from "not at all" to extremely". They will report transient stressors, including home, school, job, conflicts with partner or children, and financial stress.[22] Participants will also be queried if they used any coping mechanisms that day using

the same options in coping mechanisms questionnaire, and asked to select all that apply.

- *Acute cravings:* Cravings will be assessed using an adapted momentary scale of the Food Cravings Inventory. The FCI is 28-item questionnaire queries five different subscales including high fat foods, sweets, carbohydrates/starches, fast foods, and fruits and vegetables.[23] FCI responses are via a 5-point scale, with higher scores indicates more frequent craving. The FCI subscales were found with high reliability (0.76-0.93) and validity (0.68-0.84).[23] In the momentary question, we will ask how often they felt cravings for each of the 5-main items, with similar Likert scale responses. For example, they will be asked how frequent they craved carbohydrates/starches during the day. If they answer they did experience cravings, a subsequent question will ask if they acted on these cravings during the day.
- *Daily Physical Activity*: Participants will be asked if they engaged in moderate to vigorous physical activity, and if so, how many minutes during the day. These questions will be based off the Godin-Shepard leisure questionnaire.[24]
- *Daily Sleep*: Participants will be asked, based on the previous night, how many hours of actual sleep they received, their bed-time and wake-time, and overall sleep quality. These questions are adapted from the Pittsburgh Sleep Quality Index, which is validated in adults.[25]
- *Survey burden:* Survey burden will be assessed using a 1-item question at the end of the survey. Participants will be queried to the burden of completing the survey that day, with a scale of 2-5, with a higher score indicating it was very burdensome.
- Further detail of the prompting strategy is displayed in Table 2.

Table 2. Daily Schedule of EMA Prompts sent to participants for 14-days.

**Construct**	**Morning Survey (8a-12p)**	**Period 1 (8a -11a)**	**Period 2 (11a -2p)**	**Period 3 (2p-5p)**	**Period 4 (5p-8p)**	**Evening Survey (8p-12p)**
Sampling	Fixed	Random	Random	Random	Random	Fixed
Stress and related concepts						
Acute Stress	x	x	x	x	x	x
Coping Mechanisms	X					x
Affect		x	x	x	x	
Chaos/ Disorder		x	x	x	x	x
Context		x	x	x	x	
Health Behaviors						
Diet	x					x
Cravings	x					x
Physical Activity						x
Sleep	x					
Survey Burden						x

Participants will also be surveyed about accelerometer wear and salivary cortisol collection at beginning and end of day.

- <u>Demographics and Attendance:</u> Participants will report demographic characteristics (e.g., age, race/ethnicity, income) and previous weight loss history at baseline. Session topics and number of sessions attended will be queried through program reports at Visits 2-4.
- <u>Stress and stress-related states:</u>

- *Chronic Stress*: Chronic stress will be assessed as both stressful life events, and overall stress in the past month. First, the Brief Life Event Questionnaire will be used to assess chronic stress each 3-months (Visit 1, Visit 3, Visit 4). This questionnaire is a self-report measure to quantify presence potentially traumatic and stressful events (e.g., close family member died, major financial crisis), and whether they still think about the event a lot, a little, or not at all.[22,26] Specifically, participants will be surveyed about the presence of each event within the last 6-months, or since they completed the questionnaire. This questionnaire has demonstrated high test-retest reliability (kappa: 0.88-1.0), and high sensitivity and specificity for the previous 6-months (0.89, 0.74, respectively).[27] Second, perceived stress in the past month will be assessed using the 10-item Perceived Stress Scale. This is a scale a questionnaire about feelings and thoughts over the last month.[28]
- *Early life stress:* Early life exposure to stressful events, especially during critical periods of growth, may impact child development and later health.[29,30] We will assess adverse child experiences (ACEs) through 11 questions which query participants about events that happened prior to age 18 years old. These queries will be about family dysfunction (e.g., family member with mental illness, member in prison), and abuse (physical, verbal, etc.), and are adapted from the Behavioral Risk Factor Surveillance System ACE module, which is conducted nationwide in adults.[31] Total number of ACEs will be a potential mediator in analysis..
- *Household stress:* The Confusion, Hubbub, and Order Scale (CHAOS) is a 15-item measure assessing disturbance, organization, and noise levels in the home. It has been validated in parents of young children.[21]
- *Coping Mechanisms*: Participants will be queried about using coping mechanisms, specifically "*What have you done to cope with your stress in the past 30-days?*" Participants have 12-options, including mindfulness practices, social support, sedentary behavior, and other options. These questions are based on the National Institutes of Health Effects of COVID-19 Outbreak - Adult Questionnaire for stressful events related to COVID-19.[32,33] Use of coping mechanisms, and specific coping mechanisms used will be exploratory variables.
- *Physiological Stress*: Physiologic stress will be assessed through salivary cortisol. Participant will be provided with collection tubes at each visit, to collect 5-days of cortisol (including 2-weekend days). Participants will be asked to collect a sample of their saliva within 1-hour of waking up, around the same time at all time points, and prior to consumption of sustenance. Participants will log the exact time of their sample on a sheet and place the collection tube in a fridge until the end of the EMA protocol (~14-days) when researchers will collect the sample. This sample will then be frozen until analysis. The cortisol will be analyzed using an ELISA-based immunoassay by the Disease Model and Assessment Core at the University of Kansas Medical Center. The ELISA kit proposed has a sensitivity of 0.111ng/mL and an assay range of 0.2-10mg/ml for saliva, and a reliable intra and inter assay prediction (0.41, 0.58, respectively).[34]

- Habitual Health Behaviors and weight:
  - *Dietary Intake:* Participants will complete one food frequency questionnaire, the Diet History Questionnaire III (DHQ), at each of the visits. The DHQ is a web-based system developed by the National Cancer Institute to administer food frequency questionnaires, and has been validated in adults.[35] This food frequency questionnaire will 135 common foods and beverages they consumed over the past month, and include portion sizes. The DHQ III information will be used to calculate

Healthy Eating Index 2015 (HEI). HEI is a calorie-adjusted measure based upon meeting the Dietary Guidelines for Americans, and ranges from 0-100 with a higher score indicating better healthier dietary quality.[36]

- *Cravings:* Habitual food cravings will be assessed by the Food Cravings Inventory (FCI), which assess the cravings for specific types of foods over the past month. The FCI is 28-item questionnaire queries five different subscales including high fat foods, sweets, carbohydrates/starches, fast foods, and fruits and vegetables. FCI responses are via a 5-point scale, with higher scores indicates more frequent craving. The FCI subscales were found with high reliability (0.76-0.93) and validity (0.68-0.84).[23]
- *Physical Activity and Sleep*: Physical activity will be using an Actigraph GT3X+ accelerometer (Actigraph, LLC, Pensacola, FL) recorded at 50hz sampling rate and placed on the wrist of the nondominant arm for the entirety of the EMA protocol, including overnight and water-based activities. The GGIR algorithm will be used to detect non-wear time then equate remaining time as sleep by wear angle.[37] Days with >10 hours of awake wear and ≥20 hours of total wear time (awake and sleep) will be considered a valid day. GGIR version 1.11 will be used to process metrics using the remaining raw accelerometer data.[37-39] The Euclidian Norm Minus One (ENMO), the vector magnitude of three axes minus 1 g will be calculated and used to classify physical activity level. Activity will classified as inactive (0-39 mg), light ([LPA], 40mg ≤ ENMO <100 mg), and MVPA (ENMO ≥100 mg).[40] Due to possible random wrist movement, bouts of ≥1 minute where 80% of the activity was classified as MVPA were used as performed by others.[41] Variables will be summarized for each participant for available days (minutes/day). In addition to device-based measures, a best practice is to also include questionnaires.[42] For physical activity, participants will also complete the Godin-Shephard Leisure Time Physical Activity Questionnaire to assess physical activity within the past week. This questionnaire is validated within adults.[24] As for sleep, participants will report their recent sleep habits using the Pittsburgh Sleep Quality Index, which is validated in adults.[25] Participants will report sleep quality, sleep duration, and daytime dysfunction.
- *Physical Activity and Sedentary Behavior Context:* Physical Activity and SB context will be assessed using the Nurses Health Survey for Physical Activity questionnaire, a reliable and validated questionnaire.[43] Participants will be asked based on the past year or since they completed the questionnaire, on average how much time they spent in various activities per week and number of days they exercised. There were ten categorical responses, increasing from minutes (0, 1-4, 5-19, 20-59) to hours (1, 1-1.5, 2-3, 4-6, 7-10, and 11+). In a separate section of the questionnaire, participants were asked how many hours per week they spent sitting (options: away-from-home, watching television/ streaming [herein: screens], other), and standing/walking (options: at-work, at-home), with similar categorical responses. Responses will be multiplied by appropriate conversion factors to calculate minutes/week.
- *Weight:* Participant's height (cm) and weight (kg) will be objectively measured by a trained research assistant using standard operating procedures with a mounted stadiometer and portable scale. Body mass index (BMI, $weight/height^2$) will be calculated. Weight at Visits 2-4 will be subtracted from Visit 1 (Week-0) to calculate change in weight.

- Visit 1: At this visit (60-90 minutes), consent will be obtained by the participant prior to study procedures. These procedures will be completed on the KUMC campus, namely in the weight management clinic.
    - The participant will complete questionnaires, height and weight will be obtained, and will be fitted with an accelerometer to wear for 14-days and return in-person.
    - They will also be provided salivary cortisol kits to measure for 5-days and store in a freezer.
    - Participants will have the EMA app downloaded onto their phone, set times for text message reminders, and receive a tutorial on the app. Beginning the next day, the participant will respond to EMA prompts for 14-consecutive days.
    - After the 14-day collection period, the participant will meet with the research assistant to return salivary cortisol and the accelerometer. Given all participants are engaging in a behavioral weight loss program, these visits may correspond with their existing meeting times.
- Visit 2-3: These visits (60-90 minutes), participants will return to complete questionnaires, salivary cortisol, and fitted with an accelerometer to wear for 14-days like Visit 1. The mobile app will be updated to conduct the same EMA questions for 14-days. Similar return procedures from Visit 1 for the accelerometer and salivary cortisol will be followed.
- Visit 4: The participant will complete questionnaires, salivary cortisol, accelerometer, and EMA will be completed for a final iteration. These visits will have a similar length (60-90 minutes), and return procedures as other visits.

7.0 Data and Specimen Banking

- Clinical data includes individual demographic information and participant behaviors at each visit. At each major time point (week-0, week-6, week-12, week-26), we will collect other participant data including anthropometry (weight and height), accelerometry, questionnaires related to participant behaviors, stress, and stress-related states. Program attendance data will be collected after Visit 4. These values will be reported as tabular data and stored in a REDCap secure electronic data capture system. All subject-level clinical data described in 1A will be preserved and shared. Shared data will be deidentified, and the original data will be maintained at the investigator's institution.
- Storage: Clinical data will be stored on secure server, and only required personnel will be given access to the data.
- Repository Submission: Baseline data (defined as any data collected prior to the intervention), such as demographics and tabular clinical data that require no additional analyses will be submitted after enrollment is completed and released through the repositories after that. After the study is completed and unblinded, the study team will submit all remaining scientific data to the data repositories (NIDDK) and will update the RCT status to "complete" in clinicaltrials.gov. According to the proposed project timeline, the release of the remaining scientific data will approximately coincide with the submission of final results to clinicaltrials.gov, as mandated by FDAAA, and will occur by the end of award or publication, whichever comes sooner.

- Specimen banking: Completed salivary tubes will be frozen until analysis and stored at a biospecimen freezer at the KUMC weight management clinic. This data will be processed within 1-year of collection to calculate salivary cortisol, and remaining biospecimen will not be stored.

8.0 Sharing of Results with Subjects

Individual results, including the participant's height and weight and the average amount of physical activity and sleep at each timepoint, will be shared with participants after study completion. An aggregate summary of the study results will be shared after study completion with participants. The data collected will be used for publications and serve as preliminary data for future grant applications related to modifying stress and stress-related concepts for health behavior change.

9.0 Study Timelines

Subject's Duration: 26-weeks /6-months

Time to enroll all subjects: 3 years, December 2027

Study completion: March 2029

10.0 Inclusion and Exclusion Criteria

Web screen: Interested participants will complete a web screening tool, where they will provide their age, sex, race, and other information. Eligible participants will be contacted for a phone screen.

Phone screen: After completing the web screening tool, a research staff member will contact the patient to confirm this information and interest in the study. At the phone screen, the researcher will also inquire about inclusion criteria (medications, etc.). Study screening for eligibility criteria will be ascertained using REDCap. Participants that meet the inclusion criteria and do not have any exclusionary criteria will be asked to schedule a visit.

Inclusion criteria:

- Enrolled in a community-based weight loss program centered in a metropolitan area of a Midwest state. Based on this criteria, we will anticipate participants are greater than 18 years of age, and have obesity.
- Must own a smart phone and be willing to download the mobile application to receive text messages.

Exclusion criteria:

- Report a current medical condition or treatment for a medical condition that could affect body weight.
- Current psychological condition (such as clinically diagnosed depression) that is untreated, hospitalization for a psychological condition within the past 12 months, or not being on a stable dose of treatment for at least 6 months.

- Is not fluent in writing or speaking the English language.
- Mobility limitations so they are unable to perform physical activity.
- Family member currently enrolled in the study or has a family member that previously participated in the study.

11.0 Vulnerable Populations

This study plans to recruit from community-based behavioral weight loss programs. Research staff members will communicate to the participant that participation in this program is not required as part of enrollment in the behavioral weight loss program, nor is their continued participation required in the research study. The focus of this study is to include all responders, including weight loss program members who do not lose weight or ultimately do not continue with the program. Moreover, incentive payments are linked to their completing of research items, rather than behavior change or weight loss as part of the program.

12.0 Local Number of Subjects

This study plans to screen 150 participants to enroll 50 participants.

13.0 Recruitment Methods

- Recruitment: Prospective participants will be recruited at their initial consultation visit for their behavioral weight loss program. Flyers will also be presented to participants at their initial visit. (pending fall 2024). Will be submitted to the IRB for approval before use. We will use a study email for communication with potential and current participants (INSTANT@KUMC.edu). This study email address will have confidential level of security.
- Stipends: Participants will be paid up to $300 via their ClinCard for completing the study. Participants will earn up to $50 per visit, which includes $25 for completing 1-morning, and 2-within days surveys for 5 days, and an additional $10 for completing 7-days in a row. Completion of at least 4 salivary cortisol measures and returning the accelerometer will be another $15. Participants will receive and additional $50 for completing visits 1 & 4.
- Potential Waived initiation fee: Participants may receive a waived or discounted enrollment fee for the University of Kansas Medical Center's behavioral weight loss program, or the 5-3-2-1 program. This reduced or waived fee may translate to a discount of up to $500. This reduced or waived fee will be provided after enrollment in the study. Enrollment in the 5-3-2-1 program is not required as part of this study, and participants may still enroll in another behavioral weight loss program of their choice to meet the study requirements.
- Participant Incentives: For completing Visit 1, participants will be given fitbit/wearable devices (estimated value $100). These devices can be used during the accelerometer wear periods. However, participants will be told to prioritize wearing the accelerometer during the selected periods.

14.0 Withdrawal of Subjects

Participation is voluntary, so participants may withdraw from the study at any time. Data that are collected from the participant prior to withdrawal will be used unless specifically noted. Participants may withdraw from the study if they do not wish to cooperate with the staff.

15.0 Risks to Subjects

- Anthropometry: There are no foreseeable risks or discomforts to anthropometric measurements. These measurements are like measures taken at an average doctor visit.
- Accelerometry/ physical activity and sleep: We have found there is no more risk to wearing the activity monitor than a typical watch. There are no foreseeable risks or discomforts with the accelerometer wear. In the unlikely event that a participant experiences an injury, the assessments will be discontinued. Participants may find the accelerometer uncomfortable or bothersome to wear; however; the accelerometer is small, light, and comes with an adjustable strap to make the device as comfortable and unobtrusive as possible. Accelerometer wear over the requested for four periods across the study (0-weeks, 6-weeks, 12-weeks, and 26-weeks) may also be burdensome but our team has performed a similar procedure in this age range with no concerns. Two trained researchers will be available to properly adjust the accelerometer if there is discomfort.
- Mobile Health Application: There are no foreseeable risk or discomforts for using the mobile app. The content will be short, and created for a beginner reading level. We will disable GPS so not to collect this identifiable data. Applicable questions will have an “other” or “don’t know” option if participants do not wish to report information. All data obtained from these devices will be stored on a secure server.
- Questionnaires: There are no foreseeable risks or discomforts for completion of questionnaires. This includes the questionnaires assessing dietary intake, healthy behaviors, and daily stress. The ACEs questionnaire will query sensitive topics, and participants will be given the option to refuse if they do not wish to answer.
- Salivary cortisol: There are no foreseeable risks or discomforts for completion of salivary cortisol collection. These measures are similar to spitting out tooth paste in a sink, which participants likely perform daily, and require only small amounts of saliva.

16.0 Potential Benefits to Subjects

Participants will receive information on their physical activity and sleep which may be beneficial for engaging in a weight loss program. They may also receive a stipend for participating in the study. Benefits of participating in this study should outweigh the risks for all participants in this study.

17.0 Data Management and Confidentiality

- Power and Sample Size: Based on past EMA work (n=39), there was an association between daily morning stress and feeding practices (p=0.01).[44] Considering physical activity and sleep are also impacted by stress,[10,11] we anticipate being able to detect an association

between stress variability with physical activity and sleep with prescribed amount (*n*=50). Even accounting for a full-time job much of the day, and fewer opportunities to examine stress and behavior (at least 2 observations/day) and minimal compliance during the weekend (4 observations/day), we would still have ≥20 observations/participant (≥1,000 observations) to detect meaningful associations.

- Statistical Analysis:
  - *Specific Aim 1: Hypothesis 1a*: Non-multilevel linear regression will examine baseline and change frequency in daily stress (morning survey) with weight loss at 12-weeks and 26-weeks. *Hypothesis 1b:* Multi-level models with logit link function (and binomial distribution) will be used to examine association between stress and subsequent MVPA and sleep (same day), with an interaction of time*individual stress. For exploratory analyses, we will examine change score within the day (within day survey differences), the lagged day effects of stress (i.e., stress on day 1 influencing physical activity and sleep on day 2), and the outcome of weight loss at week-6.
  - *Specific Aim 2: Hypothesis 2*: Non-multilevel linear regression will examine baseline and change frequency in coping (morning survey) and positive affect (within day survey) with weight lost at week-12 and week-26. Potential covariates include number of ACEs, program compliance (i.e., number of sessions attended), coping mechanisms (number and type), age, and sex. Statistical significance will be set at *p*=0.05.

- This study involves minimal physical risk to participants. Nevertheless, the research team will follow all standard procedures of research at KUMC to ensure subject privacy and anonymity. This study will ask for date of birth to ensure meeting age requirements (>18 years), and this information will be stored electronically in and in secure, access-restricted folders on the KUMC network. The questions asked from the mobile health app will not contain PHI or other identifiable information. Data from the mobile health application will be stored virtually in LifeData mobile application software, which is a secure network. All mobile application data will then be stored electronically in a secure, access-restricted folder on the KUMC network. The app will be deleted off the phone after the second visit.

- Trained and qualified research staff will used to collect and enter data. These staff will be employed by the KUMC Human Energy Balance Core. The research staff will follow ALCOA (attributable, legible, contemporaneous, original, accurate) standard for documentation. REDCap will be used for data entry. The research coordinator will ensure that accurate and complete data is transcribed from the paper source into REDCap. Data entry will be done twice to maintain data quality, accuracy, and integrity. The data comparison tool will use in REDCap to produce a "Clean" final entry. The final entry will be marked complete and locked. Locked data will be ready for analysis. Additionally, the study coordinator will set up REDCap alerts, notifications, and survey auto-invitations to streamline the screening process and maintain an up-to-date contact log, ensuring efficient communication and data management throughout the study.

- Salivary cortisol: Specimens will be collected at KUMC facilities and stored at the freezer within the facility. For processing, these samples will be transported in appropriate freezer temperature to final processing destination (i.e., KUMC laboratories).

18.0 Provisions to Monitor the Data to Ensure the Safety of Subjects: NA

19.0 Provisions to Protect the Privacy Interests of Subjects

- All participant information will be coded with a study identification number to maintain confidentiality. In the case where identifiable information needs to be retained (e.g., signed consent document, etc.), this information will be kept separate from information and data in which identifiable information has been removed.
- All study data that is in paper form is secured in a locked file cabinet that is located in a locked room. Only appropriate personnel will have access to this information. Moreover, the building is secured at the close of the business day and the building also has an alarm system.
- All study data that is in electronic format is secured on institutional servers, are password protected, and have current security software and hardware installed. Only appropriate study investigators and staff are allowed access to these electronic data files. Passwords are required to be changed on a regular basis.
- If a subject indicates that they may know a member of the study staff and desires not to have direct contact with that individual, that request will be honored by the investigators.
- Subjects will be adequate time to review the study consent document and to have their questions asked to and answered by the investigators prior to providing consent.
- Prior to each procedure being performed for this study a description will be provided to the subject and the subject will have the opportunity to asks questions prior to engaging in that procedure.
- Participants can refuse to answer or skip questions in questionnaires that are validated with this response option. Participants can refuse to complete a procedure or answer specific questions unless these are needed to determine eligibility for the study Participants failing to provide response to questions or not completing other measures that are required to determine eligibility for this study will not be eligible.
- The research staff for this study will only be permitted to access subject information that is outlined in the consent document, and access to this information will be based on the specific role of the research staff member on this project, with other access not permitted.

20.0 Compensation for Research-Related Injury

We do not anticipate any research related injury due to the physical assessments in this study.

21.0 Economic Burden to Subjects

The subjects will be responsible for transportation to and from study visits. They will also be responsible for maintaining their data plan and continued use of a cellphone through the study period (26-weeks).

22.0 Consent Process

At the first visit (~60-90 minutes), the study will be described in a friendly manner, and consent will be obtained by the participant. Consent will be obtained on the KUMC campus. Study data will not be collected until consent is obtained.

23.0 Process to Document Consent in Writing

We will obtain written informed consent from the participant before study procedures.

24.0 Setting

Potential participants will be recruited from the KUMC Weight Management clinic in Kansas City area. Research procedures will be completed at research sites of the University of Kansas Medical Center. All records containing PHI, including consent forms, will be stored in subject binders in locked cabinets in the research coordinator's office with access only to the designated study team. All biospecimens will be stored in a secure area with limited access. Databases with identifying information will be encrypted and password protected. The study team will be trained in the University of Kansas Medical Center HIPAA guidelines.

25.0 Resources Available

- KUMC Clinical Weight Management (Kirmayer Building) is a 5,000 ft^2 facility, that originally served as a Sports Medicine Clinic and has been occupied by The Division of Physical Activity and Weight Management since the spring of 2012. This facility includes the following: an 800 ft^2 waiting room, 4 staff offices, an office suite for project coordinators, 4 exam rooms (~ 120 ft^2), a conference room, and an exercise room for conducting supervised aerobic or resistance exercise with adjacent handicap accessible locker rooms for both men and women, and extensive audio and video entertainment packages to facilitate participant compliance. Exercise equipment including over 100 ActiGraphs will be available for the current study.

- LifeData: is an ecological momentary assessment platform designed for researchers, and educators. This platform delivers in the moment questions via notification. Participants tap the notification allowing them to answer questions right away. Flexible scheduling options and the ability to create multiple question/schedule combinations accommodate even the most complex study designs.

REFERENCES:

1. Wang Y, Beydoun MA, Min J, Xue H, Kaminsky LA, Cheskin LJ. Has the prevalence of overweight, obesity and central obesity levelled off in the United States? Trends, patterns, disparities, and future projections for the obesity epidemic. *Int J Epidemiol*. Jun 1 2020;49(3):810-823. doi:10.1093/ije/dyz273
2. Washburn RA, Szabo-Reed AN, Gorczyca AM, et al. A Randomized Trial Evaluating Exercise for the Prevention of Weight Regain. *Obesity (Silver Spring)*. Jan 2021;29(1):62-70. doi:10.1002/oby.23022
3. Katzmarzyk PT, Martin CK, Newton RL, Jr., et al. Weight Loss in Underserved Patients - A Cluster-Randomized Trial. *N Engl J Med*. Sep 3 2020;383(10):909-918. doi:10.1056/NEJMoa2007448
4. Pigsborg K, Kalea AZ, De Dominicis S, Magkos F. Behavioral and Psychological Factors Affecting Weight Loss Success. *Curr Obes Rep*. Sep 2023;12(3):223-230. doi:10.1007/s13679-023-00511-6
5. Elfhag K, Rossner S. Who succeeds in maintaining weight loss? A conceptual review of factors associated with weight loss maintenance and weight regain. *Obes Rev*. Feb 2005;6(1):67-85. doi:10.1111/j.1467-789X.2005.00170.x
6. Dunton GF, Kawabata K, Intille S, Wolch J, Pentz MA. Assessing the social and physical contexts of children's leisure-time physical activity: an ecological momentary assessment study. *Am J Health Promot*. Jan-Feb 2012;26(3):135-42. doi:10.4278/ajhp.100211-QUAN-43
7. Joseph NT, Jiang Y, Zilioli S. Momentary emotions and salivary cortisol: A systematic review and meta-analysis of ecological momentary assessment studies. *Neurosci Biobehav Rev*. Jun 2021;125:365-379. doi:10.1016/j.neubiorev.2021.02.042
8. Weber J, Angerer P, Apolinario-Hagen J. Physiological reactions to acute stressors and subjective stress during daily life: A systematic review on ecological momentary assessment (EMA) studies. *PLoS One*. 2022;17(7):e0271996. doi:10.1371/journal.pone.0271996
9. Naya CH, Zink J, Huh J, Dunton GF, Belcher BR. Examining the same-day relationship between morning cortisol after awakening, perceived stress in the morning, and physical activity in youth. *Stress*. May 2021;24(3):338-347. doi:10.1080/10253890.2020.1804852
10. Liao Y, Shonkoff ET, Dunton GF. The Acute Relationships Between Affect, Physical Feeling States, and Physical Activity in Daily Life: A Review of Current Evidence. *Front Psychol*. 2015;6:1975. doi:10.3389/fpsyg.2015.01975
11. Mason TB, Do B, Wang S, Dunton GF. Ecological momentary assessment of eating and dietary intake behaviors in children and adolescents: A systematic review of the literature. *Appetite*. Jan 1 2020;144:104465. doi:10.1016/j.appet.2019.104465
12. Dunton GF. Ecological Momentary Assessment in Physical Activity Research. *Exerc Sport Sci Rev*. Jan 2017;45(1):48-54. doi:10.1249/JES.0000000000000092
13. Greaney JL, Darling AM, Saunders EFH, Almeida DM. Daily Stress and Microvascular Dysfunction: The Buffering Effect of Physical Activity. *Exerc Sport Sci Rev*. Jan 1 2023;51(1):19-26. doi:10.1249/JES.0000000000000310
14. Dunton GF, Crosley-Lyons R, Rhodes RE. Affective Response During Real-World Physical Activity as an Intervention Mediator. *Exerc Sport Sci Rev*. Oct 1 2023;51(4):140-149. doi:10.1249/JES.0000000000000321

15. Kracht CL, Chaput JP, Martin CK, Champagne CM, Katzmarzyk PT, Staiano AE. Associations of Sleep with Food Cravings, Diet, and Obesity in Adolescence. *Nutrients*. Nov 30 2019;11(12)doi:10.3390/nu11122899
16. Hayes JF, Schumacher LM, Panza E, Dunsiger SI, Wing RR, Unick JL. Affective responses to overeating episodes in women participating in a behavioral weight loss program. *Eat Behav*. Jan 2022;44:101599. doi:10.1016/j.eatbeh.2022.101599
17. Smith KE, Mason TB, Wang WL, et al. Dynamic associations between anxiety, stress, physical activity, and eating regulation over the course of a behavioral weight loss intervention. *Appetite*. Jan 1 2022;168:105706. doi:10.1016/j.appet.2021.105706
18. Trofholz A, Tate A, Janowiec M, et al. Ecological Momentary Assessment of Weight-Related Behaviors in the Home Environment of Children From Low-Income and Racially and Ethnically Diverse Households: Development and Usability Study. *JMIR Res Protoc*. Dec 1 2021;10(12):e30525. doi:10.2196/30525
19. Burke LE, Shiffman S, Music E, et al. Ecological Momentary Assessment in Behavioral Research: Addressing Technological and Human Participant Challenges. *J Med Internet Res*. Mar 15 2017;19(3):e77. doi:10.2196/jmir.7138
20. Dunton GF, Liao Y, Dzubur E, et al. Investigating within-day and longitudinal effects of maternal stress on children's physical activity, dietary intake, and body composition: Protocol for the MATCH study. *Contemp Clin Trials*. Jul 2015;43:142-54. doi:10.1016/j.cct.2015.05.007
21. Matheny AP, Wachs TD, Ludwig JL, Phillips K. Bringing order out of chaos: Psychometric characteristics of the confusion, hubbub, and order scale. *Journal of Applied Developmental Psychology*. 1995/07/01/ 1995;16(3):429-444. doi:https://doi.org/10.1016/0193-3973(95)90028-4
22. Berge JM, Tate A, Trofholz A, et al. Examining within- and across-day relationships between transient and chronic stress and parent food-related parenting practices in a racially/ethnically diverse and immigrant population : Stress types and food-related parenting practices. *Int J Behav Nutr Phys Act*. Jan 16 2018;15(1):7. doi:10.1186/s12966-017-0629-1
23. White MA, Whisenhunt BL, Williamson DA, Greenway FL, Netemeyer RG. Development and validation of the food-craving inventory. *Obes Res*. Feb 2002;10(2):107-14. doi:10.1038/oby.2002.17
24. Amireault S, Godin G. The Godin-Shephard leisure-time physical activity questionnaire: validity evidence supporting its use for classifying healthy adults into active and insufficiently active categories. *Percept Mot Skills*. Apr 2015;120(2):604-22. doi:10.2466/03.27.PMS.120v19x7
25. Buysse DJ, Reynolds CF, 3rd, Monk TH, Berman SR, Kupfer DJ. The Pittsburgh Sleep Quality Index: a new instrument for psychiatric practice and research. *Psychiatry Res*. May 1989;28(2):193-213. doi:10.1016/0165-1781(89)90047-4
26. Berge JM, Trofholz A, Tate AD, et al. Examining unanswered questions about the home environment and childhood obesity disparities using an incremental, mixed-methods, longitudinal study design: The Family Matters study. *Contemp Clin Trials*. Nov 2017;62:61-76. doi:10.1016/j.cct.2017.08.002
27. Brugha TS, Cragg D. The List of Threatening Experiences: the reliability and validity of a brief life events questionnaire. *Acta Psychiatr Scand*. Jul 1990;82(1):77-81. doi:10.1111/j.1600-0447.1990.tb01360.x
28. Cohen S, Kamarck T, Mermelstein R. A global measure of perceived stress. *J Health Soc Behav*. Dec 1983;24(4):385-96.

29. Hughes K, Bellis MA, Hardcastle KA, et al. The effect of multiple adverse childhood experiences on health: a systematic review and meta-analysis. *Lancet Public Health*. Aug 2017;2(8):e356-e366. doi:10.1016/S2468-2667(17)30118-4
30. Foright RM, McQuillan TE, Frick JM, et al. Exposure to early-life stress impairs weight-loss maintenance success in mice. *Obesity (Silver Spring)*. Jan 2024;32(1):131-140. doi:10.1002/oby.23931
31. Ye D, Reyes-Salvail F. Adverse childhood experiences among Hawai'i adults: Findings from the 2010 Behavioral Risk Factor Survey. *Hawaii J Med Public Health*. Jun 2014;73(6):181-90.
32. Hamilton CM, Strader LC, Pratt JG, et al. The PhenX Toolkit: get the most from your measures. *Am J Epidemiol*. Aug 1 2011;174(3):253-60. doi:10.1093/aje/kwr193
33. Krzyzanowski MC, Terry I, Williams D, West P, Gridley LN, Hamilton CM. The PhenX Toolkit: Establishing Standard Measures for COVID-19 Research. *Curr Protoc*. Apr 2021;1(4):e111. doi:10.1002/cpz1.111
34. systems BRD. Cortisol Paramter Assay Kit. https://www.rndsystems.com/products/cortisol-parameter-assay-kit_kge008b
35. Subar AF, Thompson FE, Kipnis V, et al. Comparative validation of the Block, Willett, and National Cancer Institute food frequency questionnaires : the Eating at America's Table Study. *Am J Epidemiol*. Dec 15 2001;154(12):1089-99. doi:10.1093/aje/154.12.1089
36. Panizza CE, Shvetsov YB, Harmon BE, et al. Testing the Predictive Validity of the Healthy Eating Index-2015 in the Multiethnic Cohort: Is the Score Associated with a Reduced Risk of All-Cause and Cause-Specific Mortality? *Nutrients*. Apr 5 2018;10(4)doi:10.3390/nu10040452
37. Migueles JH, Rowlands AV, Huber F, Sabia S, van Hees VT. GGIR: A Research Community–Driven Open Source R Package for Generating Physical Activity and Sleep Outcomes From Multi-Day Raw Accelerometer Data. *Journal for the Measurement of Physical Behaviour*. 01 Sep. 2019 2019;2(3):188-196. doi:10.1123/jmpb.2018-0063
38. Sabia S, van Hees VT, Shipley MJ, et al. Association between questionnaire- and accelerometer-assessed physical activity: the role of sociodemographic factors. *Am J Epidemiol*. Mar 15 2014;179(6):781-90. doi:10.1093/aje/kwt330
39. van Hees VT, Gorzelniak L, Dean Leon EC, et al. Separating movement and gravity components in an acceleration signal and implications for the assessment of human daily physical activity. *PLoS One*. 2013;8(4):e61691. doi:10.1371/journal.pone.0061691
40. Hildebrand M, VT VANH, Hansen BH, Ekelund U. Age group comparability of raw accelerometer output from wrist- and hip-worn monitors. *Med Sci Sports Exerc*. Sep 2014;46(9):1816-24. doi:10.1249/MSS.0000000000000289
41. da Silva SG, Evenson KR, da Silva ICM, et al. Correlates of accelerometer-assessed physical activity in pregnancy-The 2015 Pelotas (Brazil) Birth Cohort Study. *Scand J Med Sci Sports*. Aug 2018;28(8):1934-1945. doi:10.1111/sms.13083
42. Gill JM, Chico TJ, Doherty A, et al. Potential impact of wearables on physical activity guidelines and interventions: opportunities and challenges. *Br J Sports Med*. Aug 7 2023;doi:10.1136/bjsports-2023-106822
43. Wolf AM, Hunter DJ, Colditz GA, et al. Reproducibility and validity of a self-administered physical activity questionnaire. *Int J Epidemiol*. Oct 1994;23(5):991-9. doi:10.1093/ije/23.5.991

44. Berge JM, Fertig AR, Trofholz A, Neumark-Sztainer D, Rogers E, Loth K. Associations between parental stress, parent feeding practices, and child eating behaviors within the context of food insecurity. *Preventive Medicine Reports*. 2020/09/01/ 2020;19:101146. doi:https://doi.org/10.1016/j.pmedr.2020.101146
