# Supplementary material for: Intensive longitudinal assessment of stress and stress-related concepts across a weight loss program: Protocol for a longitudinal ecological momentary assessment study
Source: PLoS One. 2026 Sep 10;21(9):e0357572. doi: 10.1371/journal.pone.0357572 (PMC13561333; doi:10.1371/journal.pone.0357572)
Supplement: Supplementary file 3 [file pone.0357572.s002.docx]

| **Supplementary Table 2. Morning Survey Questions, Answers, and Descriptions** | | |
| --- | --- | --- |
| - Available: 1 time per day, 8:00am-12:00pm - Overview: Daily Diary Questions and Answers related to sleep, cravings, and diet from previous day - Completion: Participants can go back through survey | | |
| **Question** | **Answers** | **Description** |
| Good morning, we would like to learn more about YOUR sleep habits LAST NIGHT.  1. What time did you fall asleep last night? | DATE ____:____ AM/PM  *(note in LifeData this includes a date with time, so the participant can select times beyond 12:00am)* | The participant will list the time that they fell asleep. Fell asleep means when they actually *went* to sleep, rather than got in bed to go to sleep, which may be an earlier time. If the participant is having trouble entering in the date, make sure the time is correct. |
| 2. Did you wake up during the NIGHT? (e.g., go to the bathroom, another disturbance) | - Yes🡪 Next question - No - Don’t Know | Was there any time that the participant woke up during the night? |
| (if participant responds yes to #2)  2a. how much long were you awake LAST NIGHT (e.g., time spent to go to the bathroom or address disturbance)? | ___Hours____minutes  *(Life data scroll option for each, 0-23 hours, 0-59 minutes)* | This question asks how long they were awake for, which may range from minutes to hours. We are only counting items where you woke up for more than 5 minutes. |
| 3. What time did you wake up this morning? | DATE ____:____ AM/PM  *(note in LifeData this includes a date with time, so the participant can select times beyond 12:00am)* | Time the participant woke up in the morning. If the participant is having trouble entering in the date, make sure the time is correct. |
| 4. How much *actual* sleep do you think YOU got LAST NIGHT? *(This may be different than the number of hours you spent in bed)* | ___Hours____minutes  *(Life data scroll option for each, 0-23 hours, 0-59 minutes)* | This is the amount of time the participant was **actually** asleep, which would exclude time from the total if they woke up at night, and from when they got in the bed (which is not necessarily sleeping). |
| 5. How would you rate your sleep quality overall from LAST NIGHT? | - Very bad - Fairly bad - Fairly good - Very good | The participant should rate their quality of sleep from last night using the prompts. |
| 6. Did you wear the tracker while you were sleeping last night? | - Yes - No - Don’t Know | The participant should report if they wore the activity tracker for the full night. **If there is difficulty wearing the tracker, please contact us.** |
| 7. Did you complete the saliva collection this morning within 30 minutes of waking?   *As a reminder, the collection should also be prior to drink and food.* | - Yes - Yes, but not within 30 minutes - No - N/A completed 5 Saliva days | The participant should report if they completed the saliva collection as prescribed. If they have already completed the 5 days successfully, please select accordingly**. If there is any difficulty performing the saliva collection, please contact us.** |
| 8. These next few questions ask about your diet yesterday and coping with stress.   In what ways did you cope with stress yesterday? Select all that apply. Remember to consider actions taken during the day. | - I did not use any techniques to cope with stress - Meditation/Mindfulness - Talking with friends - Engaging in family activities - More screen activities (i.e., TV, social media) - Getting exercise - Eating more often - Reading, puzzles, cross words - Going outside - Drinking alcohol - Using tobacco or marijuana - Talking to health care provider/therapist - Other | Select the all the different ways that the participant coped with stress yesterday. If the action is performed generally and not necessarily to cope with stress, you may not need to report it. If something other than options listed, select other and explain. |
| 9. Thinking about yesterday, how often did you have cravings for:   Carbohydrates/starches (e.g., bread, rice) | - Never - Rarely (once or twice) - Sometimes - Often - Always | We are interested in how much they craved carbohydrates or starches throughout the day (including morning and night). Examples are provided, but the participant may have craved other carbohydrates/starches.  The participant can mark multiple cravings. |
| 10. Thinking about yesterday, how often did you have cravings for:   Fast food | - Never - Rarely (once or twice) - Sometimes - Often - Always | We are interested in how much they craved fast food throughout the day (including morning and night). Examples are provided, but the participant may have craved other fast food.  The participant can mark multiple cravings. |
| 11. Thinking about yesterday, how often did you have cravings for:   High fat foods (e.g., burgers, friend chicken, high fat meats) | - Never - Rarely (once or twice) - Sometimes - Often - Always | We are interested in how much they craved high fat foods throughout the day (including morning and night). Examples are provided, but the participant may have craved other high fat foods.  The participant can mark multiple cravings. |
| 12. Thinking about yesterday, how often did you have cravings for:   Sweets (e.g., cookies, ice cream) | - Never - Rarely (once or twice) - Sometimes - Often - Always | We are interested in how much they craved sweets throughout the day (including morning and night). Examples are provided, but the participant may have craved other sweets.  The participant can mark multiple cravings. |
| 13. Thinking about last night, how often did you have cravings for:   Fruits and Vegetables | - Never - Rarely (once or twice) - Sometimes - Often - Always | We are interested in how much they craved fruits and vegetables throughout the day (including morning and night). The participant can mark multiple cravings. |
| 14. Did you indulge in any cravings last night? | - Yes - No - Unsure | Did the participant eat any of the foods that they were craving last night? |
| 14 a. If yes, which ones did you indulge in? *Select all that apply* | - Carbohydrates - fast food - High fat foods - Sweets - Fruits and Vegetables | If yes, what type of food did the participant eat?  Participant can mark multiple but should only report those that were eaten in response to cravings rather than regular meal patters. |
| Thank you for answering these questions. We will now briefly ask about your current state.   15. On a scale from 0-10, with 0 being not stressed at all and 10 being very stressed, how would you rate your level of stress RIGHT NOW? | Not stressed 0  1  2  3  4  5  6  7  8  9  Very Stressed 10 | How stressed is the participant on a scale of 1-10 right now. Report how they are currently feeling prior to the prompt – rather than in response to the prompt. Higher score indicates higher stress. |
| 16. On a scale from 0-10, with 0 being totally unable and 10 being completely able, how would you rate your ability to manage stress RIGHT NOW? | Totally Unable 0  1  2  3  4  5  6  7  8  9  completely able 10 | On a ten-point scale, the participant how able they are to cope with stress right now. This response should be how they are currently feeling prior to the prompt – rather than in response to the prompt. Higher score indicates higher ability to cope with stress. |
