# Supplementary material for: Intensive longitudinal assessment of stress and stress-related concepts across a weight loss program: Protocol for a longitudinal ecological momentary assessment study
Source: PLoS One. 2026 Sep 10;21(9):e0357572. doi: 10.1371/journal.pone.0357572 (PMC13561333; doi:10.1371/journal.pone.0357572)
Supplement: Supplementary file 4 [file pone.0357572.s003.docx]

| **Supplementary Table 3. Evening Survey Questions, Answers, and Descriptions** | | |
| --- | --- | --- |
| - Available: 1 time per day, 6:00pm-10:00pm - Overview: Daily Diary Questions and Answers related to current stress and daily stressors - Completion: Participants can go back through survey | | |
| **Question** | **Answers** | **Description** |
| Good evening. We’d like to learn more about your day.   1. Overall, how stressful was your day? | - Extremely - Quite a bit - A little - Not at all | Thinking about the full day, the participant should rate how stressful it was. |
| 2. Which of these things have caused you stress today? Select all that apply. | - Job - Conflicts with Partner - Conflicts with Children - Other home conflicts - Friends - Finances - School - Other - No causes of stress today | Participants should mark all items that caused additional stress today. These may be small one-time conflicts or ongoing problems they actively dealt with today. No causes of stress should be reported when they had no other causes. |
| 3. Considering today, how much do you agree or disagree with these statements as they relate to you and your family:   **We almost always seemed to be rushed** | - Strongly disagree - Somewhat disagree - Somewhat agree - Strongly agree | These following statements are related to the participant and their current family/unit within their household, as this unit may impact their daily behaviors and stress. These questions are NOT about relatives that do not reside within their domicile or are a part of everyday items. For example, if the participants grandmother is generally late but the participant did not interact with them that day or their actions did not impact the family unit that day – they would not be considered in these responses.  This statement asks how often the participant felt they and their family were rushed or moving frantically **that day**. Participants should consider the full day (morning, noon, and night). |
| 4. Considering today, how much do you agree or disagree with these statements as they relate to you and your family:   **No matter what our family planned, it usually didn't seem to work out** | - Strongly disagree - Somewhat disagree - Somewhat agree - Strongly agree | This statement asks how often the participant felt they and their family planned something or had planned events, and it didn’t go accordingly to plan **that day**. Participants should consider the full day (morning, noon, and night). |
| 5. Considering today, how much do you agree or disagree with these statements as they relate to you and your family:   **No matter how hard we tried, we always seemed to be running late** | - Strongly disagree - Somewhat disagree - Somewhat agree - Strongly agree | This statement asks how often the participant felt they and their family were late to items during **the day**. Participants should consider the full day (morning, noon, and night). |
| 6. Considering today, how much do you agree or disagree with these statements as they relate to you and your family:   **We had regular routines/schedules in our home (e.g., teeth brushing, family meals, bedtimes, chores)** | - Strongly disagree - Somewhat disagree - Somewhat agree - Strongly agree | This statement asks how often the participant felt they and their family had regular routines occur within their home **that day.** The question provides examples, but there may be other routines that are followed within their home. Participants should consider the full day (morning, noon, and night). |
| 7. Considering today, how much do you agree or disagree with these statements as they relate to you and your family:   **You couldn't hear yourself think in your home** | - Strongly disagree - Somewhat disagree - Somewhat agree - Strongly agree | This statement asks how often the participant felt they and their family had trouble concentrating due to excess noise or talking **that day.** The noise may be due to TV/electronic noise, overlapping conversations, or other noisy distractions. Participants should consider the full day (morning, noon, and night). |
| 8. How much time did you engage in moderate activity today? Moderate activity gets your heart rate up to a high level but is not exhaustive (e.g., fast walking, tennis, easy swimming). | ___Hours____minutes  *(Life data scroll option for each, 0-23 hours, 0-59 minutes)* | Moderate activity is exercise-like intensity activity. This may be activity where the participant’s heart rate is up (or high), but you can generally speak to someone else or continue a conversation. General walking, like walking to the mailbox, may considered light activity. These activities may be daily activities, or structured. Examples are provided, but the participant may consider other activities they perform. Consider the total amount though out the day, and report that amount. |
| 9. How much time did you engage in vigorous activity today? Vigorous activity includes activities where your heart beats rapidly (e.g., running, vigorous bicycling, basketball). | ___Hours____minutes  *(Life data scroll option for each, 0-23 hours, 0-59 minutes)* | Vigorous activity is the highest exercise intensity. This is where the participant’s heart rate is up (or high), and it is difficult to continue a conversation. The participant should consider sustained activities, more than 1 minute or so, rather than difficulty walking upstairs for 20 seconds minutes a few times a day. These activities may be daily activities, or structured. Examples are provided, but the participant may consider other activities they perform |
| 10. Did you wear the tracker today? | - Yes - No - Don’t Know | The participant should report if they wore the activity tracker for the full day. **If there is difficulty wearing the tracker, please contact us.** |
| 11. Overall, how difficult was it for you to fill out the surveys TODAY? | - Not at all - A Little - Moderately - Quite a bit - Extremely | Considering the full day, the participant should consider how difficult it was to answer surveys. **If the participant is having significant problems with the prompting schedule or receiving prompts – please contact us.** |
