# Supplementary material for: Intensive longitudinal assessment of stress and stress-related concepts across a weight loss program: Protocol for a longitudinal ecological momentary assessment study
Source: PLoS One. 2026 Sep 10;21(9):e0357572. doi: 10.1371/journal.pone.0357572 (PMC13561333; doi:10.1371/journal.pone.0357572)
Supplement: Supplementary file 7 [file pone.0357572.s004.rtf]

Supplementary Table 4. Within day Survey Questions, Answers, and Descriptions	
•	Available: 4 times per day, sent randomly between 4, 3-hour windows, 8:00-11:00am, 11:00am-2:00pm, 2:00pm-5:00pm, 5:00pm-8:00pm
•	Overview: Short Survey Question and Answers related to Stress and Movement Context in the moment
•	Completion: Participants can NOT go back through survey	
Unlike the morning and evening questions, these questions are meant to be quick and focus on what the participant is feeling and doing at that moment. The participant should be prepared to answer all questions in a row, since the participant will not be able to go back.	
Question	Answers 	Description	
In these questions, we want to learn more about how YOU are doing right now. 
1.	Do you feel like things are going according to plan right now? 	•	Yes 
•	Somewhat 
•	No	The participant should consider how much they feel their day is going according to plan, either their internal planned day or actual planned events.
	
2.	Right before this prompt went off, how HAPPY were you feeling?	•	Extremely 
•	Quite a bit 
•	A little 
•	Not at all 	The participant should report how happy or elated they were feeling prior to the prompt going off. The participant should report their feelings regardless of receiving the prompt or how receiving the prompt may have influenced these feelings.	
3.	Right before this prompt went off, how FRUSTRATED/ANGRY were you feeling?	•	Extremely 
•	Quite a bit 
•	A little 
•	Not at all 	The participant should report how frustrated or angry they were feeling prior to the prompt going off. The participant should report their feelings regardless of receiving the prompt or how receiving the prompt may have influenced these feelings.	
4.	Right before this prompt went off, how STRESSED were you feeling?	•	Extremely 
•	Quite a bit 
•	A little 
•	Not at all 	The participant should report how stressed they were feeling prior to the prompt going off. The participant should report their feelings regardless of receiving the prompt or how receiving the prompt may have influenced these feelings.	
5.	Right before this prompt went off, how CALM/RELAXED were you feeling?	•	Extremely 
•	Quite a bit 
•	A little 
•	Not at all 	The participant should report how calm or relaxed they were feeling prior to the prompt going off. The participant should report their feelings regardless of receiving the prompt or how receiving the prompt may have influenced these feelings.	
6.	Right before this prompt went off, how SAD/DEPRESSED were you feeling?	•	Extremely 
•	Quite a bit 
•	A little 
•	Not at all 	The participant should report how sad or depressed they were feeling prior to the prompt going off. The participant should report their feelings regardless of receiving the prompt or how receiving the prompt may have influenced these feelings.	
7.	Where are you located?
	•	Home – Indoors 
•	Home – outdoors 
•	Someone else's home 
•	Outdoors – not at home 
•	Work 
•	Car/Van/Truck/Vehicle 
•	Childcare/School 
•	Gym/ recreation center 
•	Store/mall 
•	Restaurant 
•	Other 	The participant should report where they are when the prompt went off. If the participant is in-between options, please choose the BEST option for their location. If they do not want to report the location, they may choose “other”.	
8.	Who are you actively involved with as you are doing your current activity?
	•	No one 
•	Friends 
•	Coworkers 
•	Child(ren) 
•	Spouse/Partner 
•	Extended Family 
•	Other 	The participant should report who they were personally with as they were doing their activity. For example, if they were sitting and doing work on a computer, they may report “no one”, whereas if they were sitting at a restaurant talking with their spouse – they may report “spouse/partner”. Please select all that apply.
